# Dr. George Humphrey Goltry

**Published:** 1904-06

**Authors:** 


Dr. George Humphrey Goltry, a graduate of the University of Buffalo, class of 1860, died at his residence near Irelandville, N. Y., April 16, 1904, aged 71 years.

